# Production of medium-chain volatile flavour esters in *Pichia pastoris* whole-cell biocatalysts with extracellular expression of *Saccharomyces cerevisiae* acyl-CoA:ethanol *O*-acyltransferase Eht1 or Eeb1

**DOI:** 10.1186/s40064-015-1195-0

**Published:** 2015-09-02

**Authors:** Shiwen Zhuang, Junshu Fu, Chris Powell, Jinhai Huang, Yihe Xia, Ruixiang Yan

**Affiliations:** School of Life Sciences, Tianjin University, Tianjin, China; National Food Institute, Technical University of Denmark, 2800 Kg. Lyngby, Denmark; Division of Food Sciences, School of Biosciences, University of Nottingham, Sutton Bonington Campus, Nottinghamshire, UK

**Keywords:** Yeast, Biosynthesis, Ester, Enzyme, Integrated

## Abstract

Medium-chain volatile flavour esters are important molecules since they have extensive applications in food, fragrance, cosmetic, paint and coating industries, which determine different characteristics of aroma or taste in commercial products. Biosynthesis of these compounds by alcoholysis is catalyzed by acyl-CoA:ethanol *O*-acyltransferases Eht1 or Eeb1 in *Saccharomyces cerevisiae.* In this study, these two yeast enzymes were selected to explore their preparations as the form of whole cell biocatalysts for the production of volatile flavour esters. Here, the novel whole cell biocatalysts *Pichia pastoris* yeasts with functional extracellular expression of Eht1 or Eeb1 were constructed. Flavour production was established through an integrated process with coupled enzyme formation and ester biosynthesis in the recombinant yeasts in one pot, leading to the formation of volatile C6–C14 methyl and ethyl esters from wort medium. Interestingly, there is no significant difference between *P. pastoris*-*EHT1* and *P. pastoris*-*EEB1* in substrate preference during flavour biosynthesis, indicating a similar role of Eht1 and Eeb1 in *P. pastoris* cells, in contradiction with previous findings in *S. cerevisiae* to some extent. Consequently the study not only provides a greater understanding of these two enzymes in a heterogeneous host, but also demonstrated the positive effect of the recombinant Eht1 and Eeb1 in ester formation by *P. pastoris* live cells, potentially paving the way towards achieving efficient production of volatile flavour by an integrated biocatalytic system composed of recombinant enzyme production and flavour biosynthesis.

**Electronic supplementary material:**

The online version of this article (doi:10.1186/s40064-015-1195-0) contains supplementary material, which is available to authorized users.

## Background

Flavour compounds have a wide range of applications in food, beverage, cosmetic and pharmaceutical fields (Noguerol-Pato et al. 2012; Cheong et al. 2013; Descours et al. 2013; Madruga et al. 2013; Zamzuri and Abd-Aziz, 2013). In the global consumer-driven market, one of the challenges for manufacturers is to create products faster and cheaper while continuing to offer consumers a recognizable ‘sensory experience’. Where it is not possible to manipulate process parameters directly, this can be achieved by inducing or supplementing products with specific aromatic or flavour compounds. Indeed, manufacture of some beverages such as FAB’s (Flavoured Alcoholic Beverages) (Mosher and Johnsson 2005) involves the addition of the flavours to an alcohol base. Typically such compounds are obtained by extraction from natural resources or by chemical synthesis. Natural extraction often requires expensive process coupled with aggressive conditions while chemical synthesis is simpler and cheaper, but the products cannot be accepted as ‘natural’ (Arcos et al. 1998; Adachi and Kobayashi 2005). One development was the introduction of enzymatic and whole-cell biocatalysis. The enzymes isolated from organisms provide several benefits, such as simpler reaction apparatus, higher productivity owing to higher catalyst concentration, and simpler product purification. However, an enzyme-based catalysis always relies on enzyme reaction kinetics and enzyme stability in aqueous solutions. In addition, only enzymes that were abundantly produced by cells could be used in industrial applications.

Compared to natural extraction and chemical synthesis, whole-cell biocatalysis has been increasingly attractive as an alternative approach for production of various esters. The whole-cell biocatalysis utilises an intact microorganism as a catalyst and improved the production of enzyme biocatalysis in that the enzymes can be used without isolation, purification or immobilisation under a protective environment (Matsumoto et al. 2001), showing high stereo-selectivity and regio-selectivity (Vandamme and Soetaert 2002). It is also able to carry out multipart syntheses, whereby a combined final products can be achieved through the parallel interaction of multiple intermediates occurred in the same vessel (Brault et al. 2014). Various ester compounds have been produced using *Escherichia coli* whole-cell biocatalyst (Gao et al. 2009; Brault et al. 2014) Rodriguez et al. (2014). Despite of these, the application of high-efficiency and low-cost *E. coli* could be hampered in case where disulfide bonds and glycosylations in proteins are necessary. In order to avoid a misfolded and inactive protein, it is more efficient to switch to an eukaryotic expression system such as *Pichia pastoris*, which is considered to be better equipped to fold proteins from an eukaryotic source (Cereghino and Cregg 2000, Ahmad et al. 2014). There have been several studies on *P. pastoris* whole cells with surface-displayed lipases, leading to the synthesis of short chain flavour esters (Jin et al. 2012), fructose laurate esters (Jin et al. 2013) and myristate glucose esters (Guo et al. 2015). However, in these studies, whole cell catalysts were prepared in an independent process separated from esterification reaction, resulting in an increase in workload and energy consumption, and consequently, a simplified and integrated bioprocess is demanded. More recently, Yan et al. (2014) reported an integrated lipase production and biodiesel synthesis in a recombinant *P. pastoris* yeast, where the lipases of growing cells were simultaneously utilized to produce biodiesel in situ and in one pot. However, there has been little research that explores the possibility of ester flavour synthesis using *P. pastoris* live cells.

Medium chain volatile esters are important molecules that deliver fruity and pleasant aromas such as isoamyl acetate (banana), ethyl acetate (solvent-like aroma), phenyl ethyl acetate (flowery, roses), ethyl hexanoate (sour apple) and ethyl octanoate (sour apple) (Van Laere et al. 2008; Lorenzoni et al. 2012). Biosynthesis of these compounds by alcoholysis is catalysed by a family of acyl-CoA:ethanol *O*-acyltransferases (AEATases), which proceed the transfer of fatty acyl groups from acylglycerols or acyl-CoA derivatives to alcohols (Mason and Dufour 2000; Saerens et al. 2006). Two such enzymes have been identified in *Saccharomyces cerevisiae*, namely Eht1 and Eeb1 (gene name *EHT1* and *EEB1*, respectively) (Saerens et al. 2006). Deletion experiments have shown that the loss of gene *EEB1* resulted in a severe reduction in ethyl butanoate, ethyl hexanoate, ethyl octanoate and ethyl decanoate compared to wild-type, whereas detection of *EHT1* only resulted in minor decrease in ethyl hexanoate and ethyl octanoate relative to wild-type. In addition, deletion of both genes produced these flavours at a similar level to the eeb1Δ single detection strain, indicating Eeb1 played a comparatively major role in the enzymatic bioprocess. Furthermore, Saerens and colleagues also reported performance of recombinant Eeb1 and Eht1 purified from *E. coli* as glutathione-S-transferase (GST) fusion proteins in the same paper. It has been proposed that both recombinant enzymes exhibited esterase activity, as well as AEATases function with different substrate specificity. For the latter, GST-tagged Eeb1 displayed a preference for octanoyl-CoA as a substrate whereas GST-tagged Eht1 preferred shorter chain acyl-CoA (butyryl-CoA) as a substrate (Saerens et al. 2006). In addition to this research, the same group studied the production of ethyl esters during fermentation by analysis of process parameters (Saerens et al. 2008a, b) and gene expression levels (Saerens et al. 2008b). It has been proposed that fatty acid precursor availability rather than the AEATases enzyme activity is a major limiting factor for ethyl ester production (Saerens et al. 2008a, b). They also demonstrated certain correlations between the gene expression levels and relevant flavour compounds during high gravity brewing fermentations, where the maximum expression level of *EEB1* was only correlated with the end concentration of ethyl hexanoate while expression level of *EHT1* showed a strong negative correlation with the levels of ethyl octanoate and decanoate (Saerens et al. 2008b). More recently, Chen et al. (2014) reported an elevated production of ethyl caproate in Chinese liquor using a recombinant yeast overexpressing *EHT1* with deleted *FAA1* encoding for acyl-CoA synthetases. All these findings ultimately suggested the creation of promising recombinant strains that can be applied for medium-chain ester production by exploiting the yeast enzymes Eht1 and Eeb1.

Therefore in the present work, we selected these useful enzymes to explore their preparations as the form of whole-cell biocatalysts, and applied into volatile flavour production in situ. Specifically, recombinant *P. pastoris* yeast strains with extracellular expression of Eht1 or Eeb1 was constructed and characterised. To the best of our knowledge, this is the first example of using recombinant *P. pastoris* live cells to functionally express extracellular Eht1 or Eeb1 as whole-cell biocatalysts for flavour production.

## Methods

### Microbial strains and growth medium

All strains, plasmids and primers used in this study are listed in Table 1. Primers were designed, with the assistant of DNAMAN V6 (Lynnon, USA), to amplify genes of interest. T4 DNA lipase and *Taq*DNA polymerase were purchased from Promega (USA) and used following supplier’s recommendations. *E. coli* was grown in Luria–Bertani (LB) medium containing 1 % (w/v) Bacto tryptone, 0.5 % (w/v) yeast extract and 1 % (w/v) NaCl. Yeast cultures were incubated at 30 °C for 24 h in YPD medium (1 % yeast extract, 2 % peptone, 2 % glucose, all in w/v). Recombinant yeast was grown on BMGY (buffered glycerol-complex) medium [1 % (w/v) yeast extract, 2 % (w/v) bacteriological peptone, 100 mM sodium phosphate, 1.34 % (w/v) yeast nitrogen base without amino acids and ammonium, 4 × 10^−5%^ (w/v) biotin and 1 % (w/v) glycerol, pH 6.0] and BMMY (buffered methanol-complex) medium [1 % (w/v) yeast extract, 2 % (w/v) bacteriological peptone, 100 mM potassium phosphate, 1.34 % (w/v) yeast nitrogen base without amino acids and ammonium, 4 × 10^−5 %^ (w/v) biotin and 0.5 % (w/v) methanol, pH 6.0). All chemicals were obtained from Invitrogen (USA). 12 ºPlato wort medium was prepared using 100 % barley malt.

**Table 1:** Strains, plasmids and primers used in this study

Strain/plasmid/primer	Description	Source
Strains		
*E. coli* Top 10	A strain for high-efficiency cloning and plasmid propagation	TransGen Biotech, China
*P. pastoris* GS115	A methylotrophic yeast having a mutation in the histidinol dehydrogenase gene (*his4*)	TransGen Biotech, China
Plasmids		
pGEM-T	A vector system for cloning of PCR products	Invitrogen, USA
pPIC9 K	A *P. pastoris* expression vector carrying kanamycin resistance gene	Invitrogen, USA
Primers^a^		
P1	F: 5′-TT*CCTAGG*ATGTCAGAAGTTTCCAAATG-3′	Sangon Biotech, China
	R: 5′-TA*GCGGCCGC*GTGTGACATCATACGACTAA-3′	
P2	F: 5′-TT*CCTAGG*ATGTTTCGCTCGGGT-3′	Sangon Biotech, China
	R: 5′-AT*GCGGCCGC*AACATATTTATAAAACTAAC T-3′	

^**a**^The restriction sites are underlined.

### Plasmid and recombinant strain construction

Nucleotide sequences of *EHT1* and *EEB1* (GeneBank Accession No. GU471248 and GU471249, respectively) were obtained from *S. cerevisiae* strain A1 (Huang et al. 2010a) by PCR with primers P1 and P2, respectively (Table 1). Genomic DNA was amplified using the following PCR conditions: 94 °C for 3 min, followed by 25 cycles of 94 °C for 30 s, 55 °C for 30 s and 72 °C for 90 s, and a final extension at 72 °C for 8 min. The PCR products were digested using *AvrII* and *NotI* and inserted into the corresponding sites of pPIC9k (Table 1). After being transformed into *E. coli* Top 10, recombinant plasmids were selected on LB agar plates containing 50 μg/mL kanamycin. Correct insert orientation was confirmed by both PCR and DNA sequencing. 10 µg of the recombinant plasmid DNA was linearized with restriction enzyme *SacI* and transformed into yeast strain *P. pastoris* GS115 by electroporation. Positive transformants were selected by cultivation at 30 °C on MD agar and verified by PCR.

### Extracellular expression of recombinant Eht1 and Eeb1

A single colony of the recombinant *P. pastoris* was inoculated into 25 mL BMGY medium and cultured at 30 °C, 220 rpm for 24 h. Yeast cells were harvested by centrifugation and resuspended in a 250 mL baffled flask containing 50 mL BMMY medium. The induction period was 120 h, with addition of methanol to a final concentration of 0.5 % every 12 h. at designated time intervals, supernatant from 1 ml induced culture broth was collected by centrifugation, and analyzed by SDS-PAGE as described previously (Ausubel et al. 2002). 20 μL of the supernatant was lysed for 10 min with 5 μL 2 × SDS loading buffer and the lysates were analyzed by SDS-PAGE. Proteins were visualized by staining with Coomassie Brilliant Blue R-250. Consequently, supernatant was harvested at 120 h by centrifugation and used for measuring protein concentration and esterase activity.

### Measurement of protein concentration and esterase activity

Concentrations of the recombinant proteins were determined by reading absorbance at 280 nm and 260 nm and calculated following an empirical formula (protein concentration = 1.45 × A_280_ − 0.74 × A_260_, mg/mL) (Xu 1997).

Esterase activity was measured as described previously (Malcorps and Dufour 1992) with minor modifications. A stock solution of 100 mM pNPA (*p*-nitrophenyl acetate, Sigma, USA) was prepared in CH_2_Cl_2_ as substrate. Before initiation of the assay, 40 μL of the stock solution was added into 40 mL of buffer solution (20 mM Tris–HCl, pH 8.0, 150 mM NaCl and 0.01 % Triton-X-100). 200 μL of crude extract was then incubated with 2 mL of substrate solution in test tubes at 30 °C for 30 min. After incubation, the absorbance was taken at 405 nm in an ultraviolet–visible spectrophotometer against a blank without the protein. One unit of enzyme activity was defined as the amount of the enzyme releasing 1 μmol of pNPA per min at 30 °C, pH 8.0. Samples obtained from *P. patoris* GS115 containing empty vector were also tested as a control. The experiment was conducted in triplicate.

### Integrated enzyme production and volatile flavour biosynthesis using *P. pastoris* whole-cell biocatalysts

*P. pastoris* GS115 harboring construct pPIC9k-EHT1 or pPIC9k-EEB1 was grown in 5 mL wort at 30 °C and 200 rpm. After overnight incubation, 0.5 mL of the culture was used to inoculate 50 mL of wort medium in a 250 mL baffled flask. Yeast cells were incubated at 30 °C and 200 rpm for 24 h and subsequently induced with 0.5 % methanol. Samples were taken over a period of 12 h after induction and immediate cooled on ice. Wort and cell pellets were separated by centrifugation at 4 °C and 4,000 rpm. Wort for chromatographic analysis was stored at −20 °C until required. *P. pastoris* containing empty vector was grown under identical condition as a control.

### SPME GC–MS analysis

Each sample (8 mL) was placed in a 15 mL vial and determined using SPME (solid phase micro-extraction) coupled with GC–MS (gas chromatography-mass spectrometry). A 100 µm polydimethylsiloxane coating fiber (Supelco, USA) and a manual SPME holder (Supelco, USA) were used after preconditioning according to the manufacturer’s instruction. The samples were equilibrated at 45 °C for 30 min and subsequently the SPME fiber was introduced into the vial headspace. After 30 min, the fibre was removed from the vial and immediately inserted into the GC injection port for a 5 min sample desorption.

GC–MS was carried out using an Agilent 5890 gas chromatograph (Agilent Technologies, USA) coupled to an Agilent 5975 mass selective detector operating in electron impact mode (ionization voltage 70 eV). A HP-5Ms capillary column (length, 30 m; internal diameter, 0.25 mm; film thickness, 0.5 µm; Agilent Technologies, USA) was equipped. Injector, detector and ion source temperatures were 250, 280 and 230 °C, respectively. The injection was performed in splitless mode and helium was used as carrier gas at a flow rate of 2 mL/min. The following temperature programme was used: 4 min hold at 45 °C; increase at 5 °C/min to 180 °C; increase at 25 °C/min to 250 °C; 5 min hold at 250 °C. The results were analysed using Xcalibur software (Thermo Fisher Scientific, U.K.), operating in selected ion mode, monitoring ions *m*/*z* 15, 29, 31, 43, 45, 55, 69, 73, 75, 87, 89, 92, 99, 103, 105, 115, 117, 125, 131, 145, 160, 165 and 173.

## Results and discussion

### Gene cloning of sequence alignment of *EHT1* and *EEB1*

The genes *EHT1* and *EEB1* were cloned from a wild-type *S. cerevisiae* A1 (Huang et al. 2010a, b) and gene sequences are available in NCBI database. Here, a comparison was made between our sequences to those reported ones of *S. cerevisiae* in the database (Table 2). Although varied DNA length was observed in these sequences, alignment analysis revealed a high degree of identity and a low level of E value between these sequences, irrespective of *EHT1* or *EEB1*. A detailed sequence alignment is available in Additional file 1.

**Table 2:** Comparison of sequences reported in different *S. cerevisiae*

Gene name	Genebank accession number	Strain name	DNA length (bp)	Query cover^a^	E value^a^	Identity^a^
*EHT1*	AB012577	Kyokai No.7	2,182	100 %	0.0	99 %
	NM_001178525	S288c	1,356	82 %	0.0	99 %
	*GU471248*	A1 (used in this study)	1,651	–	–	–
*EEB1*	NM_001183909	S288c	1,455	94 %	0.0	99 %
	*GU471249*	A1 (used in this study)	1,371	–	–	–

^a^The value was derived from sequence alignment between sequences used in this study and those reported ones in NCBI database.

### Heterologous expression of Eht1 and Eeb1

Heterologous expression of the two yeast enzymes was performed using the established protein expression host *P. pastoris*, which has been used for production of recombinant proteins from an eukaryotic source, either intracellularly or extracellularly (Pringle and Hartwell 1981, Pokoj et al. 2010). The reasons lie on its capacity of performing many eukaryotic post-translational modifications, such as correct formation of disulfide bonds, glycosylation and proteolytic processing, as well as a low endotoxin content compared to a bacterial expression system (Salerno and Parks 1983; Choder 1991; Gurkan and Ellar 2005, Larsen et al. 2008; Pokoj et al. 2010; Ahmad et al. 2014).

**Fig. 1:**
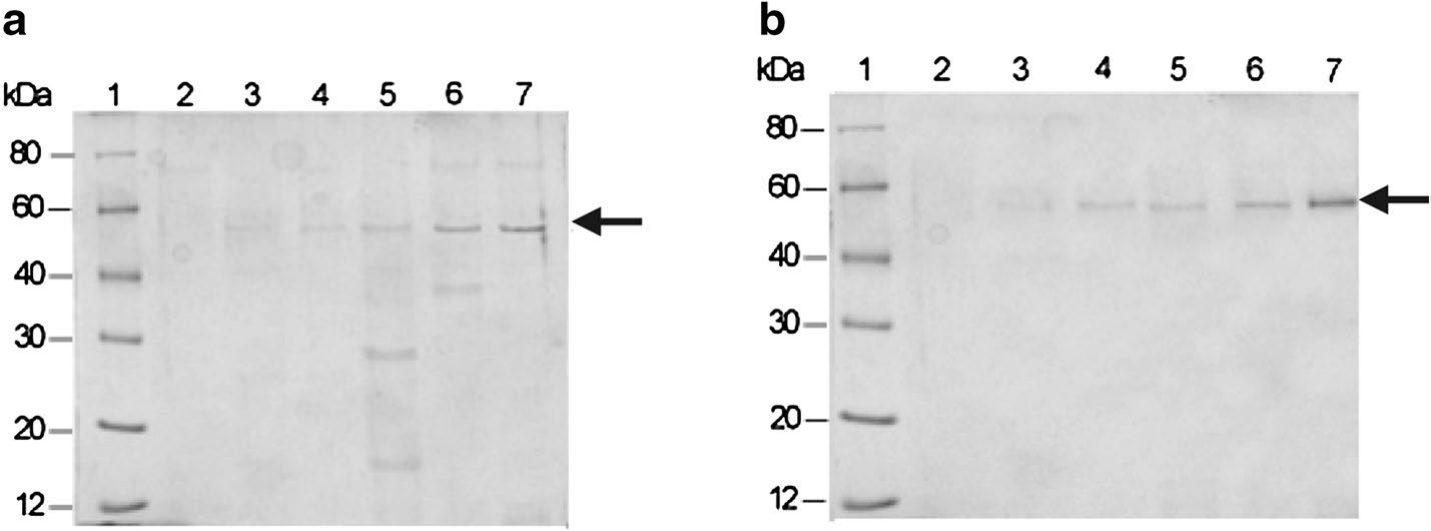
SDS-PAGE analysis of recombinant proteins expressed in *P. pastoris* GS115. *Arrows* indicate Eht1 (**a**) and Eeb1 (**b**). *Lane 1* molecular mass standards; *lane 2* culture medium of empty vector strain; *lanes 3-7* culture medium of recombinant strain induced for 24, 48, 72, 96 and 120 h, respectively. Proteins in the polyacrylamide were stained with Coomassie Brilliant Blue R-250.

**Fig. 2:**
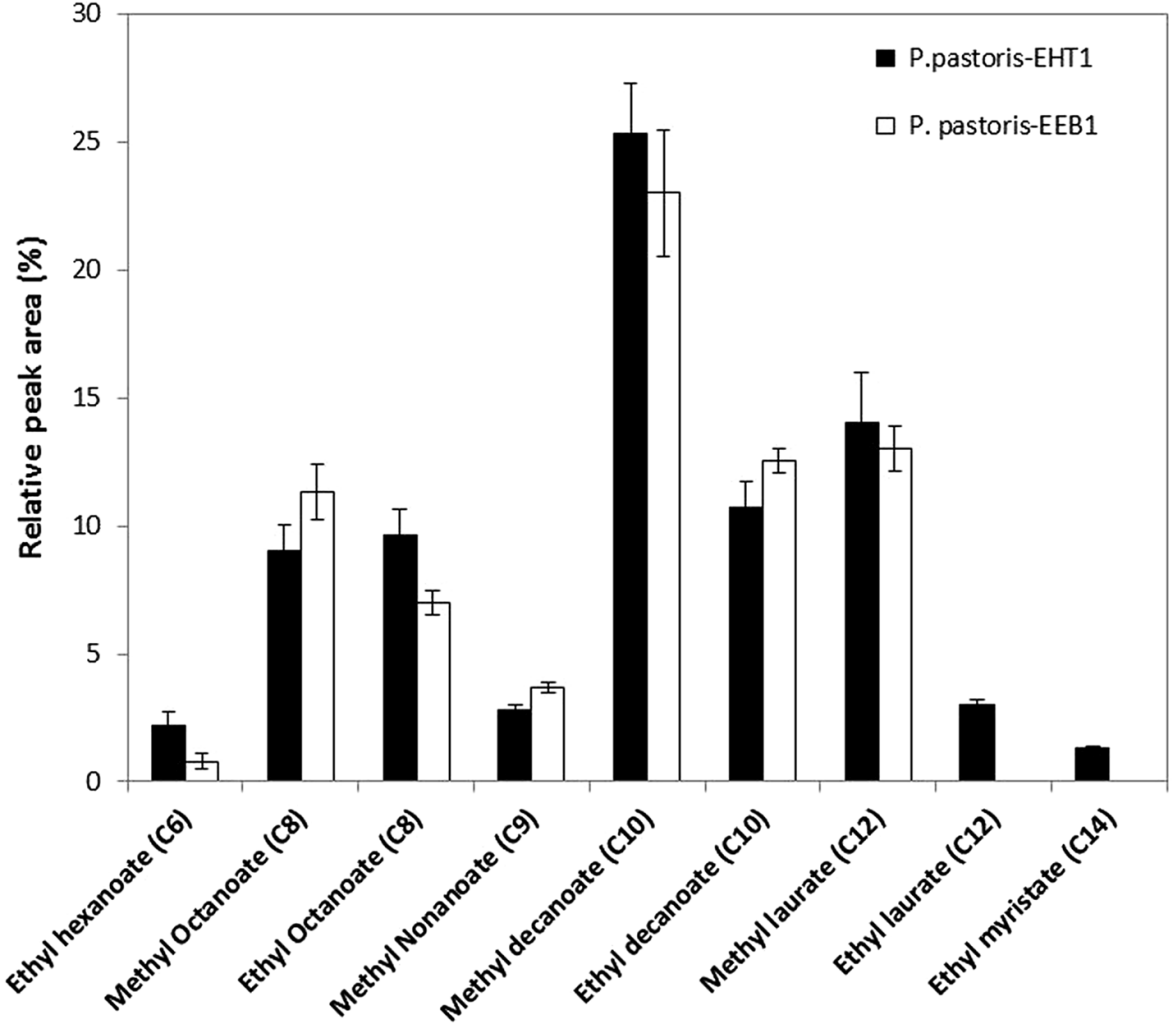
Production of volatile compounds in two recombinant *P. pastoris* strains measured by SPME GC–MS. Strains were fermented for 24 h at 30 °C in 50 mL of brewer’s wort and volatile compound production was subsequently induced with 0.5 % methanol for 12 h. *P. pastoris* harboring empty vector was fermented under identical conditions with methanol induction as a control. C6-C14 fatty acid methyl/ethyl esters were produced by the two recombinant strains but were not observed in control samples. Amounts of individual volatile compound were shown as percentage of total aromas. Data represents the mean from triplicate experiments ± standard deviation.

As shown in Fig. 1, target bands were observed to increase progressively from 24 to 120 h post-induction, irrespective of Eht1 or Eeb1, indicating the recombinant proteins were successfully expressed and secreted extracellularly. Although a native-PAGE was not performed in this study, the result also revealed that the yeast secreted very low levels of endogenous proteins as reported (Cereghino and Cregg 2000; Ahmad et al. 2014). Consequently, the recombinant Eht1 and Eeb1 displayed apparent molecular mass of 55 kDa and 56 kDa (Fig. 2), respectively, corresponding to the theoretical weight of 51 kDa and 52 kDa plus 4 kDa, likely to be due to the presence of glycosylation in the recombinant enzymes. This hypothesis was consistent with an online prediction (http://www.cbs.dtu.dk/services/NetNglyc/), where the deduced amino acid sequences of Eht1 and Eeb1 were predicted to have 4 and 2 N-linked glycosylation sites, respectively (see Additional file 2). It is known that glycosylation is one of the most common post-translational modifications of proteins in biological systems, and is one of the major advantage of *Pichia* over *E. coli.*, affecting not only the protein activities on several levels but also how the protein interacts within the biological system (Choder 1991). In future application of this work, it will be interesting to confirm this hypothesis by PAGE with glycoside hydrolase-digested samples, or by commercial glycosylation detection kits.

### The enzymatic activity of extracellular Eht1 and Eeb1

Prior to using the recombinant yeasts as whole cell biocatalysts, the 120 h post-induction supernatant containing extracellular enzymes was characterised. Consequently, the protein content was >100 mg/L and the esterase activity was >70 U/mg (normalized to the protein quantity), irrespective of the supernatant derived from *P. pastoris*-*EHT1* or *P. pastoris*-*EEB1*, whereas much lower values were detected in *P. pastoris* harboring empty vector (Table 3), again indicating the presence of extracellular enzymes produced by recombinant yeasts.

**Table 3:** Protein concentration (mg/L) and esterase activity (U/mg) of recombinant enzymes

Sample	Protein concentration (mg/L)	Esterase activity (U/mg)^a^
Recombinant Eht1^b^	100.3 ± 10.3	74.5 ± 10.5
Recombinant Eeb1^b^	113.6 ± 9.7	81.6 ± 12.7
Control^c^	7.7 ± 1.5	10.3 ± 0.7

^a^Esterase activity was determined using p-nitrophenyl acetate as a substrate; protein yield was determined based on absorbance at 280 and 260 nm. Results are expressed as the mean ± standard deviation from triplicate experiments.

^b^Sample was collected from 120 h post-induction supernatant containing extracellular enzymes by recombinant *P. pastoris.*

^c^Sample was collected from 120 h post-induction supernatant by *P. pastoris* containing empty vector.

Esterase activity of recombinant Eht1 and Eeb1 has been observed in previous studies (Saerens et al. 2006; Knight et al. 2014). Saerens et al. (2006) provided evidence that GST-tagged Eht1 and Eeb1 is able to hydrolyse p-nitrophenyl acyl esters in vitro, and interestingly Knight et al. (2014) found Eht1 was active as a thioesterase. In this study, a simplified esterase assay was performed to evaluate hydrolysis activity of the crude enzymes in the culture supernatant. It can be argued that only one substrate was used in this assay and many classes of enzymes can hydrolyze pNPacetate and therefore could explain the observed results; however, this was performed simply to verify enzymatic activity of the extracellular enzymes prior to whole-cell biocatalysis study. In future exploration of these enzymes as purified forms, various substrates should be used in this assay in order to obtain a more specific esterase activity. Consequently, the results suggested that the two yeast enzymes were produced as active extracellular forms in the engineered *P. pastoris*.

### The activity of *P. pastoris* whole-cell biocatalysts on the synthesis of volatile flavours

Saerens et al. (2006) firstly demonstrated the capacity of Eht1 and Eeb1 to synthesis medium-chain fatty acids ethyl esters in *S. cerevisiae*. In order to expand the biosynthesis of volatile flavours, we investigated the whole-cell biocatalysis model of Eht1 and Eeb1 by using recombinant *P. pastoris* live cells.

The recombinant *P. pastoris* expressing *EHT1* or *EEB1*, as well as *P. pastoris* containing empty vector (control), were cultivated on brewer’s wort. After 12 h methanol induction, formation of volatile compounds was assessed by SPME GC–MS analysis. Phenethyl alcohol was detected from all the cell cultures, indicating this compound is an endogenous metabolite derived from *P. pastoris* yeast itself from wort medium used. Apart from phenethyl alcohol, none of volatile ester compounds was observed from the control strain. For the recombinant strains, production of ethyl esters was observed to increase with the increasing chain length from C6 to C10. As shown in Fig. 2, recombinant yeast *P. pastoris*-*EHT1* produced fatty acid ethyl esters including ethyl hexanoate (C6), ethyl octanoate (C8) and ethyl decanoate (C10), corresponding to 2, 10 and 11 % of total volatiles, respectively. Similarly, during fermentation with the *P. pastoris*-*EEB1*, the levels were 1, 7 and 13 % of total volatiles for ethyl hexanoate (C6), ethyl octanoate (C8) and ethyl decanoate (C10), respectively. In addition, it is interesting to note that ethyl laurate (C12) and ethyl myristate (C14) were produced by *P. pastoris*-*EHT1*, but not by *P. pastoris*-*EEB1,* although they were only present in relatively small quantities. It should be noted that the value above was expressed in relative abundance of the gas phase, and an overall quantification of these compounds indicated that *P. pastoris*-*EEB1* produced higher amounts of esters compared to *P. pastoris*-*EHT1* expect for ethyl hexanoate (see Additional file 3), probably due to higher expression level of yeast enzyme Eeb1 extracellularly.

These results revealed that the yeast enzymes Eht1 and Eeb1 are responsible for the biosynthesis of medium-chain (C6-C14) fatty acid ethyl esters, which was consistent with the findings of Saerens et al. (2006). However, given the similar relative abundance of the compounds described above, no significant difference was observed between *P. pastoris*-*EHT1* and *P. pastoris*-*EEB1* in substrate preference during flavour biosynthesis, indicating that the two enzymes play an similar role in the yeast, resulting in a similar function of the two whole-cell biocatalysts in this study. It is surprising since the results obtained contradict to the findings of Saerens et al. (2006) to a certain extent, where genes *EHT1* and *EEB1* appear to play different roles in *S. cerevisiae*. This phenomenon could be probably due to cellular availability of relevant precursors. It is well-known that enzyme activity can be greatly influenced by the relevant precursors. Previous research involving fermentation parameters showed that fatty acid precursors such as fatty acyl-CoA play a major role for the production of ethyl esters (Saerens et al. 2008a, b). Given the basic wort medium used in this study, it is likely that there was a lack of fatty acid precursors resulting either from altered yeast metabolism or from medium composition, which will require further optimisation of the yeast or the process conditions. In addition, performance of the recombinant enzymes could be affected by the yeast metabolism within *P. pastoris* cells. Although whole-cell biocatalysis showed several advantages over isolated enzymes, cellular production can be affected by the biological environment in the cell system such as yeast secondary metabolites and intermediates.

Apart from ethyl esters, considerable methyl esters were observed in this study (Fig. 2), likely to be due to the inductive agent (methanol) of the expression system, where the yeast had priority in utilizing methanol in place of ethanol if sufficient methanol was present. In particular, either of the recombinant strain resulted in significantly higher levels of methyl decanoate. Indeed, methyl and ethyl decanoate accounts for 50.4 and 54.2 % of the total volatile aroma esters generated by *P. pastoris*-*EHT1* and *P. pastoris*-*EEB1*, respectively, indicating a high-availability of decanoyl-CoA as a substrate. Moreover, the two recombinant yeasts both produced methyl nonanoate but not ethyl nonanoate, further indicating the significance of substrate availability. More interestingly, methyl laurate was observed in both strains whereas ethyl laurate was only obtained in *P. pastoris*-*EHT1*. Given the presence of ethyl laurate is relatively low level, it is proposed that the difference between *P. pastoris*-*EHT1* and *P. pastoris*-*EEB1* was little. Furthermore, the formation of methyl esters also provides an explanation for the absence of high level ethyl esters in the volatile products. In future research it would be interesting to compare affinity of both recombinant enzymes for methanol and ethanol using biochemical in vitro tests, which could provide insights on the production of either ethyl or methyl esters.

Production of flavour esters by whole cell biocatalysts has been studied previously. For example, Jin et al. (2012) described surface immobilisation of *Candida Antarctica* lipase B (CALB) for flavour synthesis using *P. pastoris* whole cells. The CALB-displaying lipase was prepared by lyophilisation of cells prior to ester synthesis through esterification reaction. More recently, Braut et al. (2014) construct a recombinant *E. coli* expressing a newly uncovered lipase. Using this recombinant strain as s whole cell biocatalyst, flavour esters were synthesised by transesterification and esterification reactions in organic media. Similar to Jin et al. (2012), the *E. coli* whole cell biocatalyst was used as a dry powder biocatalyst. In the current study, we proposed an alternative approach of integrated flavour production. This was achieved through construction of recombinant *P. pastoris* yeast strain that was able to grow, extracellularly express Eht1 or Eeb1, and generate volatile flavour esters from wort medium simultaneously. The result offers a clue towards achieving economical production of bioflavours via simplified single-step route.

We have successfully demonstrated extracellular secretion of yeast enzymes Eht1 and Eeb1 for flavor synthesis using recombinant *P. pastoris* yeasts, but also left unanswered questions for future studies. It can be argued that these enzymes can be implicated in the intracellular metabolism and that using a secretion system is not the best way to create an efficient whole cell biocatalyst for this type of reaction. In future study it will be interesting to compare ester biosynthesis between an intracellular type of whole cell biocatalyst and the extracellular one in this study. The work would bring more interesting novelty to the yeast whole cell biocatalyst domain for flavor synthesis. Moreover, more experiments could be designed to determine the activity of both extracellular and intracellular enzymes for flavor biosynthesis in the recombinant yeasts studied. Indeed, Yan et al. (2014) constructed a recombinant *P. pastoris* yeast with extracellular expression of *Thermomyces lanuginosus* lipase for biodiesel production. The authors observed that not all lipases were secreted in recombinant *P. pastoris* yeast and consequently they demonstrated that although the intracellular lipase displayed a lower activity, they should not be underestimated for the biodiesel synthesis.

## Conclusion

In the present work, production of volatile flavour esters using recombinant *P. pastoris* live cells was investigated. The results clearly demonstrated the positive impact of extracellular expression of Eht1 and Eeb1 in flavour production by the recombinant *P. pastoris* yeasts, resulting in the formation of medium-chain fatty acid ethyl and methyl esters. Whilst more experimental work will be needed before more definitive conclusion can be offered about the efficiency of this whole-cell system, the results of our study not only provide an additional understanding of these yeast enzymes in a heterogeneous yeast, but also present an evidence of producing natural favours in an integrated system with coupled enzyme production and enzyme-catalyzed flavour synthesis in one pot.

## Additional files

**Additional file 1.** Sequence alignment for EHT1 (A) and EEB1 (B). Multiple sequence alignment was performed using online software (CLUSTAL 2.1).
**Additional file 2.** N-linked glycosylation sites prediction of Eht1 (A) and Eeb1 (B).
**Additional file 3.** Volatile ester compounds analysis by SPME GC-MS.
