# Supplementary material for: Production of medium-chain volatile flavour esters in Pichia pastoris whole-cell biocatalysts with extracellular expression of Saccharomyces cerevisiae acyl-CoA:ethanol O-acyltransferase Eht1 or Eeb1
Source: Springerplus. 2015 Sep 2;4:467. doi: 10.1186/s40064-015-1195-0 (PMC4556718; doi:10.1186/s40064-015-1195-0)
Supplement: Supplementary file 1 — Additional file 1. Sequence alignment for EHT1 (A) and EEB1 (B). Multiple sequence alignment was performed using online software (CLUSTAL 2.1). [file 40064_2015_1195_MOESM1_ESM.docx]

**Additional file 1** Sequence alignment for *EHT1* (A) and *EEB1* (B). Multiple sequence alignment was performed using online software (CLUSTAL 2.1)

1. *EHT1:* our submission (GU471248) was compared to sequences reported on NCBI data base (AB012577 and NM_001178525)

CLUSTAL 2.1 multiple sequence alignment

gi|4586631|dbj|AB012577.1| TCTTGTAAATATATGAGGTCTTTTCAAATCAATATACATAATGTTACTTA 50

gi|398365306|ref|NM_001178525. --------------------------------------------------

gi|289718294|gb|GU471248.1| --------------------------------------------------

gi|4586631|dbj|AB012577.1| TTATTTTATTCCAAGCAGGTCCATTTTTTTTTTCACTTTGCACGAAATTA 100

gi|398365306|ref|NM_001178525. --------------------------------------------------

gi|289718294|gb|GU471248.1| --------------------------------------------------

gi|4586631|dbj|AB012577.1| CATAAACATTAAAAGGACCCTTGTGTATCTGCTTAATGATTTGCAAAACC 150

gi|398365306|ref|NM_001178525. --------------------------------------------------

gi|289718294|gb|GU471248.1| --------------------------------------------------

gi|4586631|dbj|AB012577.1| TGCGGAAACGGAACGAAAAATGAGCAATCTCGTTCCGGTTCCGAGATCCT 200

gi|398365306|ref|NM_001178525. --------------------------------------------------

gi|289718294|gb|GU471248.1| --------------------------------------------------

gi|4586631|dbj|AB012577.1| TTTTTTTTTTTAAGTCTTGCGCATGTGCAGATTTTAAAAGGCGTCAGATA 250

gi|398365306|ref|NM_001178525. --------------------------------------------------

gi|289718294|gb|GU471248.1| --------------------------------------------------

gi|4586631|dbj|AB012577.1| TCGGATGTCCCTGGAAGTAATAGCTCCTCGAGTATTTCCTCCGTTTATTA 300

gi|398365306|ref|NM_001178525. --------------------------------------------------

gi|289718294|gb|GU471248.1| -----------TGGAAGTAATAGCTCCTCGAGTATTTCCTCCGTTTATTA 39

gi|4586631|dbj|AB012577.1| TTAATATGAGCAGTTTTTTAAGTTCTATTATTACATTGATAGTAGTTGCG 350

gi|398365306|ref|NM_001178525. --------------------------------------------------

gi|289718294|gb|GU471248.1| TTAATATGAGCAGTTTTTTAAATTCTATTATTACATTGATAGTAGTTGCG 89

gi|4586631|dbj|AB012577.1| TAAAAAACAAAGCTCATAAAAGTTTCCGATATGTCAGAAGTTTCCAAATG 400

gi|398365306|ref|NM_001178525. ------------------------------ATGTCAGAAGTTTCCAAATG 20

gi|289718294|gb|GU471248.1| TAAAAAACAAAGCTCATAAAAGTTTCCGATATGTCAGAAGTTTCCAAATG 139

********************

gi|4586631|dbj|AB012577.1| GCCAGCAATCAACCCATTCCATTGGGGATACAATGGTACAGTTTCGCATA 450

gi|398365306|ref|NM_001178525. GCCAGCAATCAACCCATTCCATTGGGGATACAATGGTACAGTTTCGCATA 70

gi|289718294|gb|GU471248.1| GCCAGCAATTAACCCATTCCATTGGGGATACAATGGTACAGTTTCGCATA 189

********* ****************************************

gi|4586631|dbj|AB012577.1| TTGTCGGTGAAAATGGTTCCATTAAACTCCATTTAAAAGACAACAAGGAG 500

gi|398365306|ref|NM_001178525. TTGTCGGTGAAAATGGTTCCATTAAACTCCATTTAAAAGACAACAAGGAG 120

gi|289718294|gb|GU471248.1| TTGTCGGTGAAAATGGTTCCATTAAACTCCATTTAAAAGACAACAAGGAG 239

**************************************************

gi|4586631|dbj|AB012577.1| CAAGTTGATTTTGACGAGTTCGCTAACAAATATGTCCCAACGTTGAAGAA 550

gi|398365306|ref|NM_001178525. CAAGTTGATTTTGACGAGTTCGCTAACAAATATGTCCCAACGTTGAAGAA 170

gi|289718294|gb|GU471248.1| CAAGTTGATTTTGACGAGTTCGCTAACAAATATGTCCCAACGTTGAAGAA 289

**************************************************

gi|4586631|dbj|AB012577.1| TGGTGCCCAATTCAAATTGAGTCCTTACTTGTTCACAGGTATTTTGCAAA 600

gi|398365306|ref|NM_001178525. TGGTGCCCAATTCAAATTGAGTCCTTACTTGTTCACAGGTATTTTGCAAA 220

gi|289718294|gb|GU471248.1| TGGTGCCCAATTCAAATTGAGTCCTTACTTGTTCACAGGTATTTTGCAAA 339

**************************************************

gi|4586631|dbj|AB012577.1| CTTTGTACTTAGGTGCTGCTGATTTCTCTAAGAAATTTCCTGTATTCTAC 650

gi|398365306|ref|NM_001178525. CTTTGTACTTAGGTGCTGCTGATTTCTCTAAGAAATTTCCTGTATTCTAC 270

gi|289718294|gb|GU471248.1| CTTTGTACTTAGGTGCTGCTGATTTCTCTAAGAAATTTCCTGTATTCTAC 389

**************************************************

gi|4586631|dbj|AB012577.1| GGCAGGGAAATTGTCAAATTCTCGGATGGTGGAGTTTGCACCGCTGACTG 700

gi|398365306|ref|NM_001178525. GGCAGGGAAATTGTCAAATTCTCGGATGGTGGAGTTTGCACCGCTGACTG 320

gi|289718294|gb|GU471248.1| GGCAGGGAAATTGTCAAATTCTCGGATGGTGGAGTTTGCACCGCTGACTG 439

**************************************************

gi|4586631|dbj|AB012577.1| GCTCATAGATTCATGGAAAAAGGATTATGAATTCGATCAAAGTACTACGA 750

gi|398365306|ref|NM_001178525. GCTCATAGATTCATGGAAAAAGGATTATGAATTCGATCAAAGTACTACGA 370

gi|289718294|gb|GU471248.1| GCTCATAGATTCATGGAAAAAGGATTATAAATTCGATCAAAGTACTACGT 489

**************************** ********************

gi|4586631|dbj|AB012577.1| GCTTTGATAAAAAAAAATTTGATGAAGACGAGAAGGCGACACATCCAGAA 800

gi|398365306|ref|NM_001178525. GCTTTGATAAAAAAAAATTTGATAAAGACGAGAAGGCGACACATCCAGAA 420

gi|289718294|gb|GU471248.1| GCTTTGATAAAAAAAAATTTGATGAAGACGAGAAGGCGACACATCCAGAA 539

*********************** **************************

gi|4586631|dbj|AB012577.1| GGATGGCCTCGTTTACAACCACGTACAAGGTACCTGAAAGATAATGAGTT 850

gi|398365306|ref|NM_001178525. GGATGGCCTCGTTTACAACCACGTACAAGGTACCTGAAAGATAATGAGTT 470

gi|289718294|gb|GU471248.1| GGATGGCCTCGTTTACAACCACGTACAAGGTACCTGAAAGATAATGAGTT 589

**************************************************

gi|4586631|dbj|AB012577.1| GGAAGAACTACGGGAGGTTGATCTACCCCTAGTAGTTATTCTACATGGTC 900

gi|398365306|ref|NM_001178525. GGAAGAACTACGGGAGGTTGATCTACCCCTAGTAGTTATTCTACATGGTC 520

gi|289718294|gb|GU471248.1| GGAAGAACTACGGGAGGTTGATCTACCCCTAGTAGTTATTCTACATGGTC 639

**************************************************

gi|4586631|dbj|AB012577.1| TTGCTGGTGGTAGTCATGAGCCGATTATAAGATCTCTTGCTGAAAACCTG 950

gi|398365306|ref|NM_001178525. TTGCTGGTGGTAGTCATGAGCCGATTATAAGATCTCTTGCTGAAAACCTG 570

gi|289718294|gb|GU471248.1| TTGCTGGTGGTAGTCATGAGCCGATTATAAGATCTCTTGCTGAAAACCTG 689

**************************************************

gi|4586631|dbj|AB012577.1| TCTCGCAGTGGGAGATTTCAAGTGGTCGTCCTAAATACCAGAGGTTGTGC 1000

gi|398365306|ref|NM_001178525. TCTCGCAGTGGGAGATTTCAAGTGGTCGTCCTAAATACCAGAGGTTGTGC 620

gi|289718294|gb|GU471248.1| TCTCGCAGTGGGAGATTTCAAGTAGTCGTCCTAAATACCAGAGGTTGTGC 739

*********************** **************************

gi|4586631|dbj|AB012577.1| ACGTTCCAAAATTACCACCAGAAATTTATTTACAGCTTATAACACAATGG 1050

gi|398365306|ref|NM_001178525. ACGTTCCAAAATTACCACCAGAAATTTATTTACAGCTTATCACACAATGG 670

gi|289718294|gb|GU471248.1| ACGTTCCAAAATTACCACCAGAAATTTATTTACAGCTTATCACACAATGG 789

**************************************** *********

gi|4586631|dbj|AB012577.1| ATATTCGCGAGTTTTTGCAAAGAGAAAAGCAAAGACATCCAGATAGAAAA 1100

gi|398365306|ref|NM_001178525. ATATTCGCGAGTTTTTGCAAAGAGAAAAGCAAAGACATCCAGATAGAAAA 720

gi|289718294|gb|GU471248.1| ATATTCGCGAGTTTTTGCAAAGAGAAAAGCAAAGACATCCAGATAGAAAA 839

**************************************************

gi|4586631|dbj|AB012577.1| CTATACGCTGTGGGATGCTCTTTTGGTGCTACGATGCTGGCAAACTATCT 1150

gi|398365306|ref|NM_001178525. CTATACGCTGTGGGATGCTCTTTTGGTGCTACGATGCTGGCAAACTATCT 770

gi|289718294|gb|GU471248.1| CTATACGCTGTGGGATGCTCTTTTGGTGCTACGATGCTGGCAAACTATCT 889

**************************************************

gi|4586631|dbj|AB012577.1| GGGAGAAGAGGGCGATAAATCACCTTTATCCGCAGCTGCTACTTTGTGCA 1200

gi|398365306|ref|NM_001178525. GGGAGAAGAGGGCGATAAATCACCTTTATCCGCAGCTGCTACTTTGTGCA 820

gi|289718294|gb|GU471248.1| GGGAGAAGAGGGCGATAAATCACCTTTATCCGCAGCTGCTACTTTGTGCA 939

**************************************************

gi|4586631|dbj|AB012577.1| ATCCTTGGGATCTTCTCCTTTCAGCAATTAGGATGAGCCAGGATTGGTGG 1250

gi|398365306|ref|NM_001178525. ATCCTTGGGATCTTCTCCTTTCAGCAATTAGGATGAGCCAGGATTGGTGG 870

gi|289718294|gb|GU471248.1| ATCCTTGGGATCTTCTCCTTTCAGCAATTAGGATGAGCCAGGATTGGTGG 989

**************************************************

gi|4586631|dbj|AB012577.1| TCAAGAACTTTATTTTCCAAAAATATTGCGCAATTCTTAACAAGAACCGT 1300

gi|398365306|ref|NM_001178525. TCAAGAACTTTATTTTCCAAAAATATTGCGCAATTCTTAACAAGAACCGT 920

gi|289718294|gb|GU471248.1| TCAAGAACTTTATTTTCCAAAAATATTGCGCAATTCTTAACAAGAACCGT 1039

**************************************************

gi|4586631|dbj|AB012577.1| TCAGGTTAATATGGGTGAATTAGGAGTTCCAAATGGCTCTCTCCCCGATC 1350

gi|398365306|ref|NM_001178525. TCAGGTTAATATGGGTGAATTAGGAGTTCCAAATGGCTCTCTCCCCGATC 970

gi|289718294|gb|GU471248.1| TCAGGTTAATATGGGTGAATTAGGAGTTCCAAATGGCTCTCTCCCCGATC 1089

**************************************************

gi|4586631|dbj|AB012577.1| ATCCTCCCACAGTCAAGAATCCATCTTTCTATATGTTCACGCCTGAAAAT 1400

gi|398365306|ref|NM_001178525. ATCCTCCCACAGTCAAGAATCCATCTTTCTATATGTTCACGCCTGAAAAT 1020

gi|289718294|gb|GU471248.1| ATCCTCCCACAGTCAAGAATCCATCTTTCTATATGTTCACGCCTGAAAAT 1139

**************************************************

gi|4586631|dbj|AB012577.1| CTAATAAAGGCAAAGAGCTTTAAATCGACCCGGGAATTTGATGAAGTGTA 1450

gi|398365306|ref|NM_001178525. CTAATAAAGGCAAAGAGCTTTAAATCGACCCGGGAATTTGATGAAGTGTA 1070

gi|289718294|gb|GU471248.1| CTAATAAAGGCAAAGAGCTTTAAATCGACCCGGGAATTTGATGAAGTGTA 1189

**************************************************

gi|4586631|dbj|AB012577.1| CACTGCGCCTGCTTTAGGCTTCCCAAATGCTATGGAGTATTATAAAGCGG 1500

gi|398365306|ref|NM_001178525. CACTGCGCCTGCTTTAGGCTTCCCAAATGCTATGGAGTATTATAAAGCGG 1120

gi|289718294|gb|GU471248.1| TACTGCGCCTGCTTTAGGCTTCCCAAATGCTATGGAGTATTATAAAGCGG 1239

*************************************************

gi|4586631|dbj|AB012577.1| CCAGCTCAATAAACAGAGTTGATACAATTCGGGTTCCTACCCTTGTTATC 1550

gi|398365306|ref|NM_001178525. CCAGCTCAATAAACAGAGTTGATACAATTCGGGTTCCTACCCTTGTTATC 1170

gi|289718294|gb|GU471248.1| CCAGCTCAATAAACAGAGTTGATACAATTCAGGTTCCTACCCTTGTTATC 1289

****************************** *******************

gi|4586631|dbj|AB012577.1| AATTCCAGGGATGATCCTGTTGTCGGCCCAGATCAACCATACTCAATCGT 1600

gi|398365306|ref|NM_001178525. AATTCCAGGGATGATCCTGTTGTCGGCCCAGATCAACCATACTCAATCGT 1220

gi|289718294|gb|GU471248.1| AATTCCAGGGATGATCCTGTTGTCGGCCCAGATCAACCATACTCAATCGT 1339

**************************************************

gi|4586631|dbj|AB012577.1| GGAAAAGAATCCTCGTATTTTGTATTGTAGAACCGATTTAGGTGGTCATT 1650

gi|398365306|ref|NM_001178525. GGAAAAGAATCCTCGTATTTTGTATTGTAGAACCGATTTAGGTGGTCATT 1270

gi|289718294|gb|GU471248.1| GGAAAAGAATCCTCGTATTTTGTATTGTAGAACCGATTTAGGTGGTCATT 1389

**************************************************

gi|4586631|dbj|AB012577.1| TAGCTTACCTAGATAAAGACAACAACTCGTGGGCTACCAAGGCAATTGCA 1700

gi|398365306|ref|NM_001178525. TAGCTTACCTAGATAAAGACAACAACTCGTGGGCTACCAAGGCAATTGCA 1320

gi|289718294|gb|GU471248.1| TAGCTTACCTAGATAAAGACAACAACTCGTGGGCTACCAAGGCAATTGCA 1439

**************************************************

gi|4586631|dbj|AB012577.1| GAATTTTTCACTAAGTTTGATGAATTAGTCGTATGATGTCACACAATTTT 1750

gi|398365306|ref|NM_001178525. GAATTTTTCACTAAGTTTGATGAATTAGTCGTATGA-------------- 1356

gi|289718294|gb|GU471248.1| GAATTTTTCACTAAGTTTGATGAATTAGTCGTATGATGTCACACAATTTT 1489

************************************

gi|4586631|dbj|AB012577.1| CAAACCACTTTCCTATGTATTTATACAACATTGGATCAATGCCCCTTTAT 1800

gi|398365306|ref|NM_001178525. --------------------------------------------------

gi|289718294|gb|GU471248.1| CAAACCACTTTCCTATGTATTTATACAATATTGGATCAATGCCCCTTTAT 1539

gi|4586631|dbj|AB012577.1| TTTAAGTTATGTATTTTTTGTTGTCTTCAGTATACCTAAAATTTAAAGTT 1850

gi|398365306|ref|NM_001178525. --------------------------------------------------

gi|289718294|gb|GU471248.1| TTTAAGTTATGTATTTTTTGTTGTCTTCAGTATACCTAAAATTTAAAGTT 1589

gi|4586631|dbj|AB012577.1| TTTTTCCCTATGCAGTGATTTTTATCTATATATTTTACTTTTCGTTAATT 1900

gi|398365306|ref|NM_001178525. --------------------------------------------------

gi|289718294|gb|GU471248.1| TTTTTCCCTATGCAGTGATTTTTATCTATATATTTTACTTTTCGTTAATT 1639

gi|4586631|dbj|AB012577.1| GAAGAGAGGAATTCATTCCATAAGTCCTCCTTAACTTTGAAAGTATGAGT 1950

gi|398365306|ref|NM_001178525. --------------------------------------------------

gi|289718294|gb|GU471248.1| GAAGAGAGGAAT-------------------------------------- 1651

gi|4586631|dbj|AB012577.1| ACCTCTCTCAGGAAATGTCCTATCCTCTACACTTGCAATGTACTCCTTCA 2000

gi|398365306|ref|NM_001178525. --------------------------------------------------

gi|289718294|gb|GU471248.1| --------------------------------------------------

gi|4586631|dbj|AB012577.1| AGCCCTGAGTGGCAATATCTGTCATATTTACAGCTTGCTTCACAAATTTT 2050

gi|398365306|ref|NM_001178525. --------------------------------------------------

gi|289718294|gb|GU471248.1| --------------------------------------------------

gi|4586631|dbj|AB012577.1| GGGACAGAATCGCCTTGCATCCCGAGAAGGTCAGATATAACTAGAACTTG 2100

gi|398365306|ref|NM_001178525. --------------------------------------------------

gi|289718294|gb|GU471248.1| --------------------------------------------------

gi|4586631|dbj|AB012577.1| TCCACTGGTACCGTTACCTGCACCGATACCTATTGTTGGTACTGAGAGTT 2150

gi|398365306|ref|NM_001178525. --------------------------------------------------

gi|289718294|gb|GU471248.1| --------------------------------------------------

gi|4586631|dbj|AB012577.1| TAGATGTTATGAACTGAGCCATCTTATGGGGG 2182

gi|398365306|ref|NM_001178525. --------------------------------

gi|289718294|gb|GU471248.1| --------------------------------

1. *EEB1:* our submission (GU471249) was compared to sequences reported on NCBI data base (NM_001183909)

CLUSTAL 2.1 multiple sequence alignment

gi|296148561|ref|NM_001183909. -----------ATGTTTCGCTCGGGTTACTATCCAACTGTCACTCCTAGC 39

gi|289718296|gb|GU471249.1| TTGTCATTTTAATGTTTCGCTCGGGTTACTATCCAACTGTCACTCCTAGC 50

***************************************

gi|296148561|ref|NM_001183909. CATTGGGGCTATAACGGCACCGTCAAACATGTACTGGGAGAGAAAGGAAC 89

gi|289718296|gb|GU471249.1| CATTGGGGCTATAACGGCACCGTCAAACATGTACTGGGAGAGAAAGGAAC 100

**************************************************

gi|296148561|ref|NM_001183909. CAAGTCTTTGGCTTTCAGAGATTCTAAGCGCCAAATTCCTCTTCATGAAT 139

gi|289718296|gb|GU471249.1| CAGGTCTTTGGCTTTCAGAGATTCTAAGCGCCAAATTCCTCTTCATGAAT 150

** ***********************************************

gi|296148561|ref|NM_001183909. TCGTAACCAAACACGTGCCTACACTCAAAGATGGTGCAAATTTTAGGTTA 189

gi|289718296|gb|GU471249.1| TCGTAACCAAACACGTGCCTACACTCAAAGATGGTGCAAATTTTAGGCTA 200

*********************************************** **

gi|296148561|ref|NM_001183909. AACAGCTTGCTTTTCACAGGTTACTTACAGACCCTTTATTTGTCAGCCGG 239

gi|289718296|gb|GU471249.1| AACAGCTTGCTTTTCACAGGTTACTTACAGACCCTTTATTTGTCAGCCGG 250

**************************************************

gi|296148561|ref|NM_001183909. TGATTTTTCCAAAAAATTTCAGGTATTTTATGGAAGAGAGATTATAAAAT 289

gi|289718296|gb|GU471249.1| TGATTTTTCCAAAAAATTTCAGGTATTTTATGGAAGAGAGATTATAAAAT 300

**************************************************

gi|296148561|ref|NM_001183909. TTTCTGATGGAGGGGTCTGCACTGCAGATTGGGTTATGCCTGAATGGGAG 339

gi|289718296|gb|GU471249.1| TTTCTGATGGAGGGGTCTGCACTGCAGATTGGGTTATGCCTGAATGGGAG 350

**************************************************

gi|296148561|ref|NM_001183909. CAAACATATTCATTAAATGCTGAAAAGGCTAGTTTTAACGAGAAACAATT 389

gi|289718296|gb|GU471249.1| CAAACATATTCATTAAATGCTGAAAAGGCTAGTTTTAACGAGAAACAATT 400

**************************************************

gi|296148561|ref|NM_001183909. TTCGAACGATGAAAAGGCCACCCATCCAAAGGGTTGGCCTCGTCTTCACC 439

gi|289718296|gb|GU471249.1| TTCGAACGATGAAAAGGCCACCCATCCAAAGGGTTGGCCTCGTCTTCACC 450

**************************************************

gi|296148561|ref|NM_001183909. CGAGAACTAGATATCTATCCTCTGAGGAACTTGAAAAATGTCATTCTAAG 489

gi|289718296|gb|GU471249.1| CAAGAACTAGATATCTATCCTCCGAGGAACTTGAAAAATGCCATTCTAAG 500

* ******************** ***************** *********

gi|296148561|ref|NM_001183909. GGTTATTCTTACCCTTTAGTGGTAGTACTTCATGGTCTTGCAGGGGGAAG 539

gi|289718296|gb|GU471249.1| GGTTATTCTTACCCTTTAGTGGTAGTACTTCATGGTCTTGCAGGGGGGAG 550

*********************************************** **

gi|296148561|ref|NM_001183909. CCATGAGCCTCTCATCAGGGCATTATCTGAAGATCTATCAAAAGTCGGTG 589

gi|289718296|gb|GU471249.1| CCATGAGCCTCTCATCAGGGCATTATCTGAAGATCTATCAAAAGTCGGTG 600

**************************************************

gi|296148561|ref|NM_001183909. ACGGTAAATTCCAAGTGGTAGTCCTTAATGCTAGAGGTTGCTCAAGATCT 639

gi|289718296|gb|GU471249.1| ACGGTAAATTCCAAGTGGTAGTCCTTAATGCTAGAGGTTGCTCAAGATCT 650

**************************************************

gi|296148561|ref|NM_001183909. AAAGTCACCACCCGTAGAATCTTTACCGCATTACACACAGGTGATGTGAG 689

gi|289718296|gb|GU471249.1| AAAGTCACCACCCGTAGAATCTTTACCGCATTACACACAGGTGATGTGAG 700

**************************************************

gi|296148561|ref|NM_001183909. AGAATTTTTGAACCACCAAAAAGCTCTATTTCCGCAAAGAAAAATATATG 739

gi|289718296|gb|GU471249.1| AGAATTTTTGAACCACCAAAAAGCTCTATTTCCGCAAAGAAAAATATATG 750

**************************************************

gi|296148561|ref|NM_001183909. CAGTCGGAACATCATTTGGAGCAGCCATGCTAACGAACTACTTAGGTGAA 789

gi|289718296|gb|GU471249.1| CAGTCGGAACATCATTTGGAGCAGCCATGCTAACGAACTACTTAGGTGAA 800

**************************************************

gi|296148561|ref|NM_001183909. GAAGGAGATAACTGTCCATTAAATGCTGCAGTCGCTCTCTCAAACCCATG 839

gi|289718296|gb|GU471249.1| GAAGGAGATAACTGTCCATTAAATGCTGCAGTCGCTCTCTCCAACCCATG 850

***************************************** ********

gi|296148561|ref|NM_001183909. GGATTTCGTACACACTTGGGACAAGTTGGCTCATGATTGGTGGTCCAACC 889

gi|289718296|gb|GU471249.1| GGATTTCGTACACACTTGGGACAAGTTGGCTCATGATTGGTGGTCCAACC 900

**************************************************

gi|296148561|ref|NM_001183909. ATATTTTTTCTAGAACTTTAACACAATTTCTAACAAGGACTGTCAAGGTT 939

gi|289718296|gb|GU471249.1| ATATTTTTTCTAGAACTTTAACACAATTTCTAACAAGGACTGTCAAGGTT 950

**************************************************

gi|296148561|ref|NM_001183909. AATATGAATGAGTTGCAGGTACCAGAGAATTTTGAGGTTTCGCACAAACC 989

gi|289718296|gb|GU471249.1| AATATGAATGAGTTGCAGGTACCAGAGAATTTTGAGGTTTCGCACAAACC 1000

**************************************************

gi|296148561|ref|NM_001183909. GACAGTTGAGAAACCAGTCTTTTATACGTATACCAGAGAAAATTTGGAAA 1039

gi|289718296|gb|GU471249.1| GACAGTTGAGAAACCAGTCTTTTATACGTATACCAGAGAAAATTTGGAAA 1050

**************************************************

gi|296148561|ref|NM_001183909. AGGCTGAAAAATTTACAGACATATTAGAATTCGATAATCTTTTCACTGCC 1089

gi|289718296|gb|GU471249.1| AGGCTGAAAAATTTACAGACATATTAGAATTCGATAATCTTTTCACTGCC 1100

**************************************************

gi|296148561|ref|NM_001183909. CCATCGATGGGCCTGCCGGACGGGTTAACATATTATAGAAAGGCCAGTTC 1139

gi|289718296|gb|GU471249.1| CCATCGATGGGCCTGCCGGACGGGTTAACATATTATAGAAAGGCTAGTTC 1150

******************************************** *****

gi|296148561|ref|NM_001183909. GATAAATAGATTGCCTAATATCAAAATCCCAACTTTAATAATCAACGCAA 1189

gi|289718296|gb|GU471249.1| GATAAATAGATTGCCTAATATCAAAATCCCAACTTTAATAATCAACGCAA 1200

**************************************************

gi|296148561|ref|NM_001183909. CGGATGATCCAGTTACAGGTGAAAACGTAATTCCGTACAAACAAGCTAGA 1239

gi|289718296|gb|GU471249.1| CGGATGATCCAGTTACAGGTGAAAACGTAATTCCGTACAAACAAGCTAGA 1250

**************************************************

gi|296148561|ref|NM_001183909. GAAAATCCTTGTGTGTTGTTATGCGAGACTGACTTGGGTGGACATTTGGC 1289

gi|289718296|gb|GU471249.1| GAAAATCCTTGTGTGTTGTTATGCGAGACTGACTTGGGTGGACATTTGGC 1300

**************************************************

gi|296148561|ref|NM_001183909. TTATCTCGACAATGAAAGTAATTCCTGGTTAACAAAGCAAGCTGCCGAGT 1339

gi|289718296|gb|GU471249.1| TTATCTCGACAATGAAAATAATTCCTGGTTAACAAAGCAAGCTGCCGAGT 1350

***************** ********************************

gi|296148561|ref|NM_001183909. TCTTGGGCAGCTTTGATGAGTTAGTTTTATAA------------------ 1371

gi|289718296|gb|GU471249.1| TCTTGGGTAGCTTTGATGAGTTAGTTTTATAAATATTTTCTTAGATAAAA 1400

******* ************************

gi|296148561|ref|NM_001183909. --------------------------------------------------

gi|289718296|gb|GU471249.1| TTCCTTACACATAATAATCTGTTCTTTTATTTCTCTATTTACCATCCTCC 1450

gi|296148561|ref|NM_001183909. -----

gi|289718296|gb|GU471249.1| CCACG 1455
