# Supplementary figures and images for: Production of medium-chain volatile flavour esters in Pichia pastoris whole-cell biocatalysts with extracellular expression of Saccharomyces cerevisiae acyl-CoA:ethanol O-acyltransferase Eht1 or Eeb1
Source: Springerplus. 2015 Sep 2;4:467. doi: 10.1186/s40064-015-1195-0 (PMC4556718; doi:10.1186/s40064-015-1195-0)

**Additional file 2** N-linked glycosylation sites prediction of Eht1 (A) and Eeb1 (B)

(A)


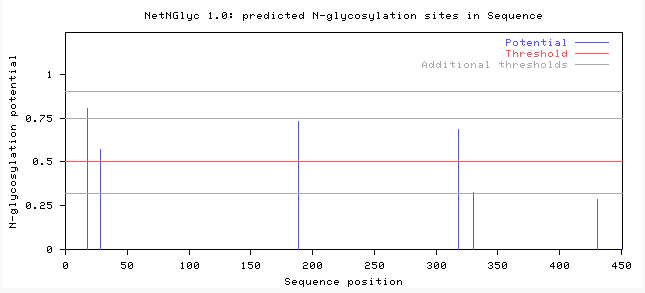


(B)

**
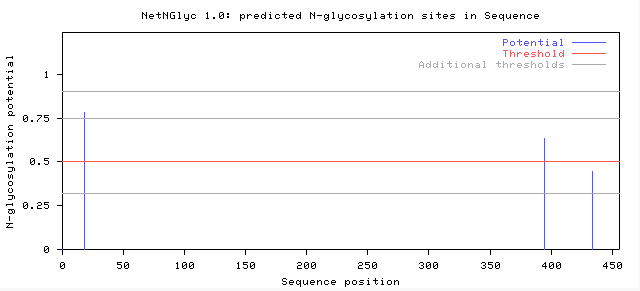
**

Supplement: Supplementary file 2 — Additional file 2. N-linked glycosylation sites prediction of Eht1 (A) and Eeb1 (B). [file 40064_2015_1195_MOESM2_ESM.docx]
