# Supplementary material for: Production of medium-chain volatile flavour esters in Pichia pastoris whole-cell biocatalysts with extracellular expression of Saccharomyces cerevisiae acyl-CoA:ethanol O-acyltransferase Eht1 or Eeb1
Source: Springerplus. 2015 Sep 2;4:467. doi: 10.1186/s40064-015-1195-0 (PMC4556718; doi:10.1186/s40064-015-1195-0)
Supplement: Supplementary file 3 — Additional file 3. Volatile ester compounds analysis by SPME GC-MS. [file 40064_2015_1195_MOESM3_ESM.docx]

**Additional file 3** Volatile ester compounds analysis by SPME GC-MS

| Compounds | Retention time (min) | Abundance | |
| --- | --- | --- | --- |
|  |  | *P. pastoris-EHT1* | *P. pastoris-EEB1* |
| Ethyl hexanoate | 11.9 | 10195 | 5600 |
| Methyl Octanoate | 15.9 | 41257 | 79268 |
| Methyl Octanoate | 17.9 | 44000 | 49000 |
| Methyl Nonanoate | 18.5 | 12756 | 25606 |
| Methyl decanoate | 21.5 | 115701 | 161000 |
| Ethyl decanoate | 24.1 | 49011 | 87822 |
| Methyl laurate | 26.1 | 64126 | 91182 |
| Ethyl laurate | 28.2 | 13620 | nd |
| Ethyl myristate | 29.8 | 5975 | nd |

nd: not detected
